# Novel calcaneal plate versus traditional philos plate for treating split fractures of humeral greater tuberosity

**DOI:** 10.3389/fsurg.2023.1272887

**Published:** 2024-01-09

**Authors:** Fei Wang, Xiaohui Niu, Haibo Xia, Wei Liang, Zhen Hu, Jun Lan

**Affiliations:** Department of Orthopaedics, Lishui City People’s Hospital, The Sixth Affiliated Hospital of Wenzhou Medical University, The First Affiliated Hospital of Lishui University, Lishui, Zhejiang, China

**Keywords:** split fractures, humeral greater tuberosity, calcaneal anatomical locking plate, proximal humeral internal locking system (PHILOS), surgery

## Abstract

**Purpose:**

To compare the effect of two internal fixation methods of calcaneal plate fixation and Philos plate fixation in treating split fractures of humeral greater tuberosity.

**Patients and methods:**

A total of 37 patients with split fractures of humeral greater tuberosity were retrospectively analyzed from September 2016 to April 2021. Enrolled patients were divided into Group A (calcaneal anatomical locking plates), and Group B [Proximal Humeral Internal Locking System (PHILOS)]. The demographics, injury-related variables, surgery-related variables, Constant-Murley score, and postoperative complication between the two groups were compared.

**Results:**

There were 16 in Group A and 21 in Group B. Fracture union was achieved in all patients, with an average of 11.9 months. The mean length of incision was significantly different between groups (Group A, 6.16 cm ± 1.07 cm; Group B, 9.09 cm ± 1.30 cm, *p* < 0.05). Significant difference was observed by comparing bleeding loss between Group A and Group B (Group A, 45.41 ± 11.19 ml; Group B, 106.06 ± 11.12 ml, *p* < 0.05). After 6 months of treatment, the average Constant-Murley score of Group A was significantly higher than that of Group B (*p* < 0.05). In terms of operation, no significant difference was observed between groups. In addition, two patients had shoulder impingement syndrome in Group B, and none in Group A.

**Conclusion:**

Calcaneal anatomical locking plate fixation is superior to Philos plate fixation in surgical trauma and bleeding loss. Our study provides an alternative technique for treating split fractures of humeral greater tuberosity.

## Introduction

Fractures of the humeral greater tuberosity occur typically in young patients with high-energy trauma, especially in males (1–3), accounting for about 20% of proximal humeral fractures (4, 5). There are many surgical treatment options for split fracture of the humeral greater tuberosity, including suture fixation, screw and washer fixation, and locking plate fixation. However, the optimal treatment of the split fractures of humeral greater tuberosity remains controversial. Currently, open reduction and internal fixation with Proximal Humeral Internal Locking System (PHILOS) is a common technique for proximal humerus fractures. However, PHILOS is not appropriate for fractures of the humeral greater tuberosity due to its hard-to-modify shape, big surface area, and size. Due to the special anatomical structure and movement characteristics at and around the greater tuberosity of the humerus, there are common reports of internal fixation failure and impingement syndrome caused by insufficient and unreasonable fixation (6, 7). Thus, according to the morphological characteristics of the proximal humerus bone, we innovatively used calcaneal anatomical locking plates to treat split fracture of the humeral greater tuberosity.

We hypothesized that a calcaneal anatomical locking plate can adequately stabilize split fracture of humeral greater tuberosity and prevent the complication of internal fixation failure and impingement syndrome.The aim of this study is to present the calcaneal anatomical locking plate fixation for split fractures of humeral greater tuberosity and evaluate its clinical outcomes.

## Materials and methods

### Patients

From September 2016 to April 2021, a total of 37 patients with split fractures of humeral greater tuberosity were treated in our hospital. Patients with fracture and dislocation underwent a routine manual reduction in emergency, and were given symptomatic treatment such as limb immobilization, detumescence and analgesia after admission. The interval from injury to operation ranged from 1 to 7 days. All operations were performed by the same surgical team. This retrospective study was approved by the institutional review board of Lishui City People's Hospital. All participants' written informed consent were obtained before its commencement. We reported this study in accordance with the Helsinki Declaration and following the Strengthening the Reporting of Cohort Studies in Surgery (STROCSS).

Inclusion criteria were as follows: (1) patients aged >18 years old; (2) patients with split fracture of the humeral greater tuberosity according to the classification for greater tuberosity fractures of the proximal humerus proposed by Mutch et al. (8) in 2014; (3) the displacement of the main bone mass of the greater tuberosity >5 mm or the angulation >45° according to preoperative CT; and (4) patients with postoperative follow-up >10 months. Exclusion criteria were as follows: (1) patients with old fracture, or pathological fracture, or open fracture; (2) patients with Bankart injury or Hill-sachs injury as well as ipsilateral nerve and vascular injury; (3)patients with other chronic diseases that affected the function of the ipsilateral shoulder joint; and (4) patients with a previous history of surgery around the ipsilateral shoulder joint.

### Surgical techniques

**Figure 1:**
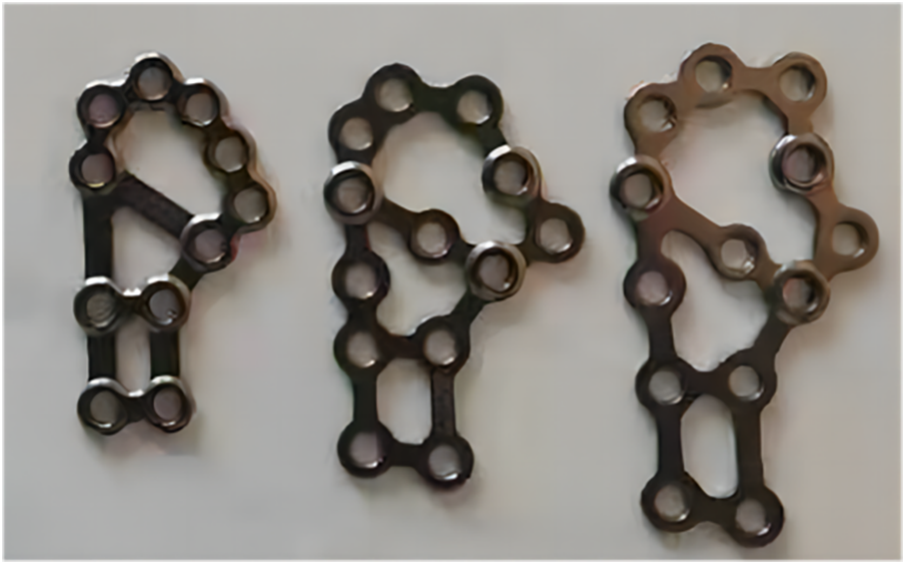
Calcaneal plates of different sizes.

**Figure 2:**
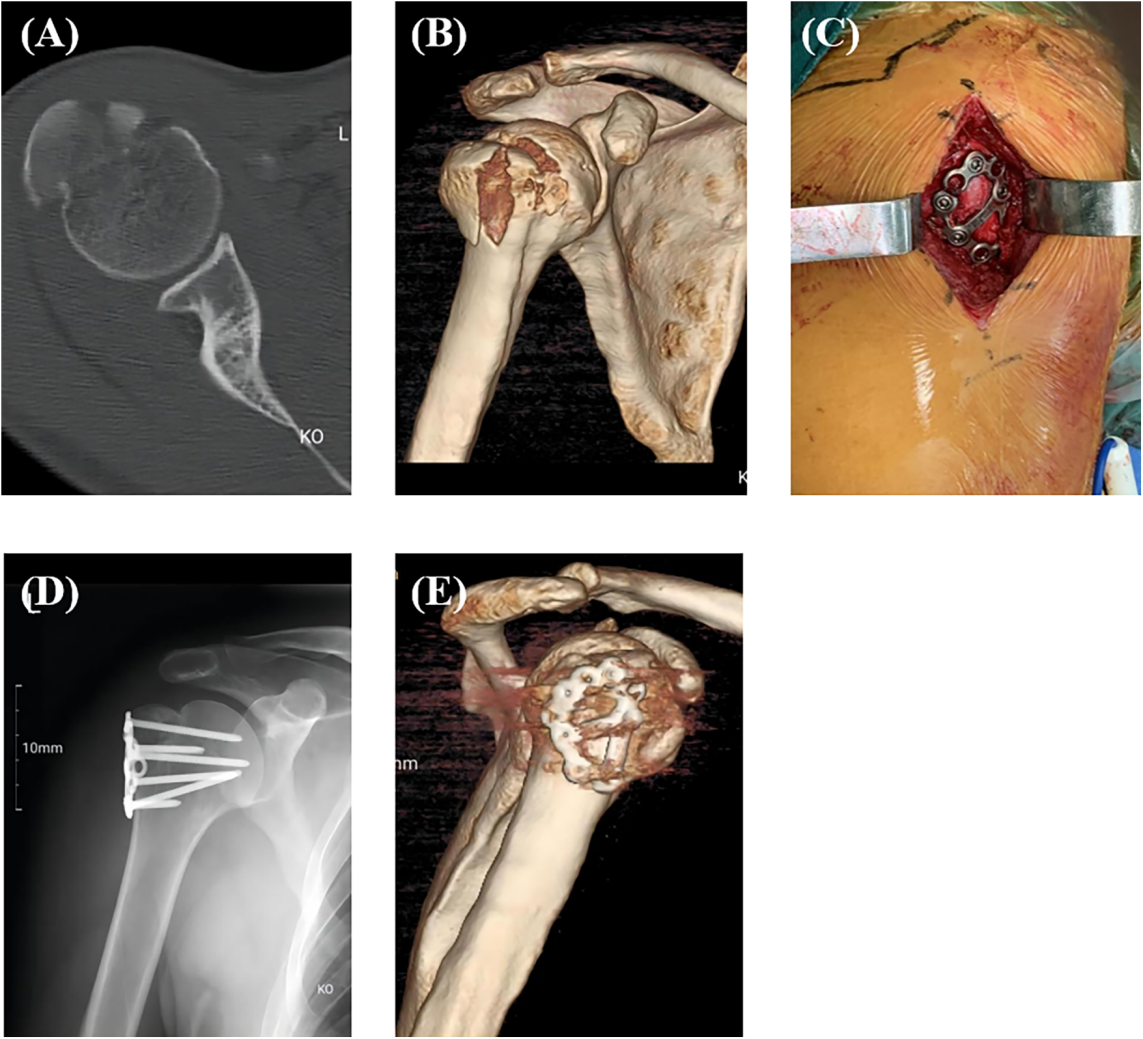
A typical case in group A. Preoperative CT (**A**) and 3D reconstruction (**B**) of the split fracture of humeral greater tuberosity; Macroscopic picture after placement of the calcaneal anatomical plate (**C**); Postoperative x-ray (**D**) and 3D reconstruction (**E**).

After successful general anesthesia, patients were adjusted to keep their beach chair position with the shoulder of the affected side padded. In Group A, the longitudinal incision was made from the inferoanterior part of the acromion (deltoid space approach). The standard thoracic deltoid approach was used in Group B. Attention was paid to careful separation during the operation to avoid damaging the axillary nerve and the feeding vessels of the humeral head. The fracture end of the greater tuberosity of the humerus was explored and cleaned to avoid soft tissue embedding, followed by the exposure and exploration of the rotator cuff insertion by rotating the shoulder joint. Rotator cuff suture traction combined with bone tenaculum can be used to reduce the fracture end, and then one or two 1.5 mm Kirschner wires were used to temporarily fix the fracture end. After the identification of the good position of the fracture end under C-arm fluoroscopy, the calcaneal plate was trimmed to cut off the excess part according to the size of the fracture fragments in Group A, followed by plastic processing and then implantation. Different sizes of anatomical plates are available (Figure 1). In Group B, the PHILOS plate was inserted via the interpectoralis major and deltoid groove approaches. The implantation position of the plate was that the upper edge should not exceed 3 mm below the apex of the greater tuberosity, and the leading edge should be about 5 mm outside the intertubercular sulcus. Screwing was performed according to the shape and position of the fracture. According to the rotator cuff injury, the injury was repaired with a non-absorbable suture, which can be sutured on the edge of the plate or screw hole through the bone-plate gap. Finally, the fracture reduction, internal fixation position and length were confirmed to be satisfactory under x-ray. The shoulder joint was mobilized to avoid subacromial impingement, followed by routine placement of the drainage rubber and suture of the incision layer by layer. A typical case in Group A was shown in Figure 2.

### Postoperative rehabilitation

After the operation, patients were informed to use the forearm sling for 4–6 weeks initially, with elbow, wrist, and finger activities within the allowable range. The shoulder joint can swing and restrict to internal rotation to protect the fracture fragment. Patients were permitted to have passive activities 1–2 weeks after operation. According to the results of imaging examination at 4 weeks after operation, the fracture healing was judged, and active assistance and active mobilization were gradually started. Weight-bearing activities could be performed 10–12 weeks after operation. In addition, patients could choose to take out the implant 12 months after operation.

### Assessments

Basic clinical information, injury-related variables, interval from injury to operation, incision length, operation time, bleeding volume and postoperative Constant-Murley shoulder joint function score, and postoperative complication were recorded. The Constant-Murley shoulder joint function score was used for evaluation of shoulder joint activity function. including pain scale 15 points, muscle strength 25 points, joint range of motion 40 points, and daily life 20 points (9). The higher the score, the better the shoulder joint function. Postoperative complications mainly include axillary nerve injury, fracture reduction loss, bone nonunion, postoperative infection, ischemic osteonecrosis, shoulder impingement syndrome, and others.

### Statistical analysis

The SPSS 22 software was used for all statistical analyses. was used to compare the variables in two groups. Quantitative variables were shown in the form of mean ± standard deviation and independent sample *t* test was used for the comparison. Categorical variables were presented in the form of frequency (%) and a χ^2^ test was used for analyses. *p* < 0.05 meant that the difference was statistically significant.

## Results

Sixteen patients were treated with calcaneal anatomic locking plate (Group A) and 21 with Proximal Humeral Internal Locking System (PHILOS) (Group B) for internal fixation. There were 24 males and 13 females, with an average age of 46.7 years old (ranging from 25 to 67). In terms of the causes of injury, there were 21 cases of falls and sports injuries, and 16 cases of traffic accident injuries. According to the preoperative CT and MRI results, 22 cases were accompanied by anterior dislocation of the glenohumeral joint, and 17 cases had fresh rotator cuff injury (Table 1).

**Table 1:** Comparison of demographics and injury-related variables between two groups.

Groups	Classifications	Group A (*n* = 16)	Group B (*n* = 21)	*p*
Gender	Males	11	13	0.666
	Females	5	8	
Age (year)		50.51 ± 12.28	48.35 ± 12.71	0.606
Cause of injury	Sports injury	10	11	0.538
	Traffic accident	6	10	
Anterior dislocation of glenohumeral join^a^	Yes	8	14	0.306
	No	8	7	
Fresh rotator cuff injury^a^	Yes	7	10	0.815
	No	9	11	

^a^ Preoperative CT and MRI results.

**Figure 3:**
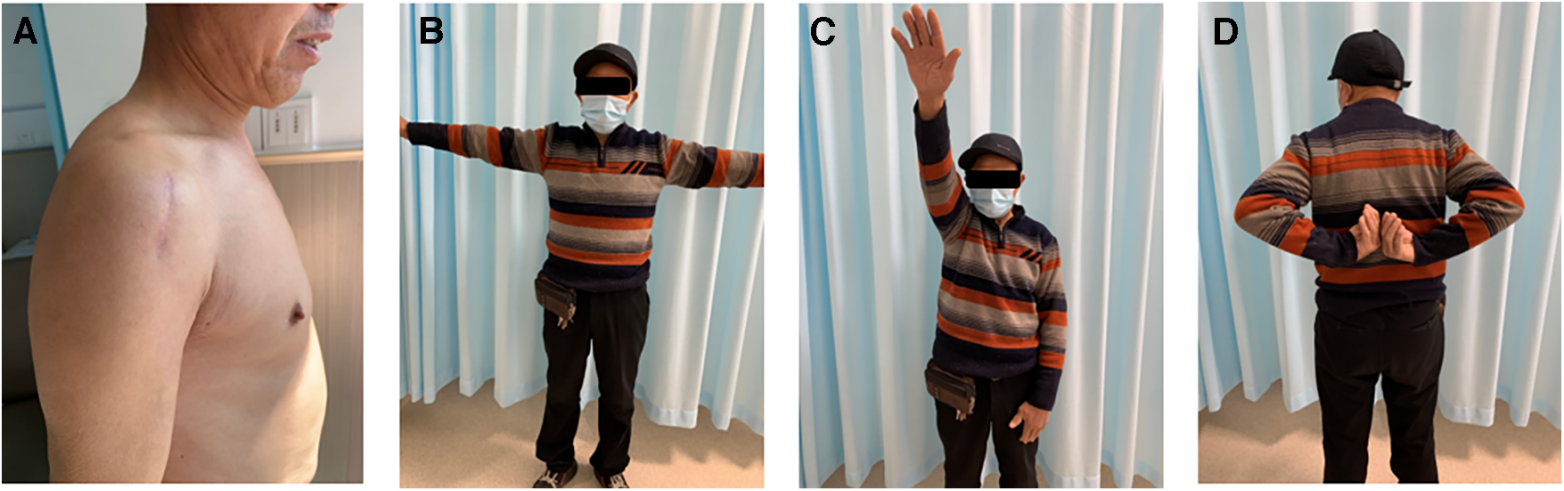
One case in group A with good shoulder function at 10 months after operation. Small surgical incision (**A**); Shoulder abduction (**B**); Shoulder flexion (**C**); Shoulder external rotation (**D**).

All 37 cases had fracture healing during the follow-up period (ranging from 10 to 23 months), with an average of 11.9 months. No significant difference was observed in the average interval from injury to operation between Group A and Group B. The average length of incision in Group A was significantly shorter than that of Group B. There was no significant difference between the average operation time of the two groups (*p* = 0.403). There was a significant difference in the comparison of bleeding loss between Group A and Group B (Group A, 45.41 ± 11.19 ml; Group B, 106.06 ± 11.12 ml, *p* < 0.05). At the last follow-up, patients in Group A showed better Constant-Murley scores than those in Group B (Group A, 87.94 ± 5.70;Group B, 79.81 ± 8.62, *p* < 0.05). One case in Group A with satisfactory shoulder function at 10 months after surgery were shown in Figure 3. No complication occurred in Group A, In Group B, two cases had shoulder impingement syndromes without secondary rotator cuff tears, and their symptoms were relieved 8 months after the removal of internal fixations (Table 2).

**Table 2:** Comparison of surgery-related variables, constant-murley score and postoperative complication between two groups.

Groups	Group A (*n* = 16)	Group B (*n* = 21)	*p*
Interval from injury to operation (day)	3.75 ± 0.45	4.14 ± 0.73	0.066
Operation time (min)	57.56 ± 7.39	60.24 ± 10.85	0.403
Incision length (cm)	6.16 ± 1.07	9.09 ± 1.30	**<0** **.** **001**
Bleeding volume (ml)	45.41 ± 11.19	106.06 ± 11.12	**<0** **.** **001**
Constant-murley score	87.94 ± 5.70	79.81 ± 8.62	**0** **.** **002**
Postoperative complication			0.204
Yes	0	2	
No	16	19	

Bold values indicate significant *p* values < 0.05.

## Discussion

Approximately 90% of the fractures of the greater tuberosity of the humerus had no or slight displacement and can be managed conservatively (10). However, split fractures of humeral greater tuberosity have different characteristics to other proximal humeral fractures. Thus, treatment for split fractures of humeral greater tuberosity should be different to that of other proximal humeral fractures (11). Although indications for surgical treatment of split fractures of humeral greater tuberosity remain controversial, the most common surgical criterion is the displacement of the main bone fragment >5 mm and/or the angulation >45° (3, 5). Some scholars even suggested that for young and active patients who need to engage in professional sports activities, the surgical indication can be expanded to the displacement of about 3 mm (12).

At present, there are multiple therapeutic choices for the surgical treatment of split fractures of humeral greater tuberosity. Arthroscopic and open approach treatments have been proposed and applied clinically, including transosseous suture fixation, tension band wires, anchors, simple screws, various plate fixation, and arthroscopic double-row suture anchors. However, it is still disputed with regard to the optimal treatment of displaced fractures (13, 14). For avulsion fractures with small fracture fragments, arthroscopic or minimally invasive small incision with double-row suture anchors, or transosseous suture and tension band internal fixation can be used (8). Furthermore, internal fixation with 4.5 mm cancellous bone screws and washers may be a simple and rapid choice for isolated fractures of the greater tuberosity. However, if there are multiple fracture fragments in the greater tuberosity, screw fixation alone cannot achieve good functional results. Simple screws may not preserve and stabilize the bone fragments in the comminuted fracture, which may lead to further aggravation of fracture fragments. Screws combined with washers may cause protrusion of the implant, which may further increase the risk of secondary impingement (15–17). Besides, for patients with fractures of the greater tuberosity accompanied by rotator cuff injuries, additional anchors are still needed for rotator cuff injury repair. It is questionable concerning the strength of anchor fixation in patients with comminuted fractures. Nevertheless, the most popular technique in the past 15 years has still been open reduction and internal fixation with plates and locking screws on the basis of its advantages in providing good biomechanical and anatomical stability (18).

At present, PHILOS has been widely used in the surgical treatment of split fractures of humeral greater tuberosity (12). Because the PHILOS plate is thick, rigid and difficult to plastic, it is not a good match for the fixation of split fractures of humeral greater tuberosity. In addition, the traditional anatomical locking plate of the proximal humerus is designed with the proximal directional screw inserted into the humeral head. The proposed directional and non-adjustable insertion trajectory cannot achieve adequate fixation for various split fracture types of humeral greater tuberosity, especially for comminuted fracture types. Occasionally, implantation of this anatomical plate in a more proximal position to capture more fracture fragments may increase the risk of shoulder impingement (19). As proposed by many scholars, various mini-locking plates could be used to treat fractures of the greater tuberosity (14, 20). Those low-profile mesh-like plates are advantageous for fractures of greater tuberosity. They cannot only adjust the implantation position according to the distribution of fracture fragments (14, 21), but also reduce the risk of shoulder impingement syndrome. Besides, the application of these plates has the advantages of small incision and less bleeding compared with the traditional PHILOS plates. However, these mini-plates often used clinically have a generally smaller size, which is difficult to cover and fix all fracture fragments perfectly. In this regard, two or more plates are commonly required for combined fixation intraoperatively, which reduces the overall stability. For patients who require simultaneous rotator cuff injury repair, it is often necessary to use additional anchors for repair due to the limitation of this type of plate. For instance, Zeng et al. (22) designed a new anatomical locking plate for fractures of humeral greater tuberosity, which can capture and fix the fracture fragments satisfactorily. Additionally, this new plate provides better repair of the rotator cuff through its separate suture hole. Because these specially designed plates are not available in most countries and regions, it is of interest to find a suitable alternative type of plate.

By reviewing the advantages and disadvantages of various implants and the local anatomical characteristics of the humeral greater tuberosity, we found that calcaneal plate is one of the best options for the treatment of split fractures of humeral greater tuberosity. To be specific, compared with the traditional proximal humeral locking plate, the calcaneal mesh anatomical locking plate has a lower profile, better elasticity and thinner thickness, which can facilitate intraoperative plastic processing and trimming. Considering the anatomical morphology of the humeral greater tuberosity, the contralateral calcaneal plate may have better compatibility and stability after pre-springing and plastic processing. Different sizes of plates are available to cover and fix fractures at any part of the humeral greater tuberosity, especially for comminuted fractures. Owing to the small size of the calcaneal plate, it is feasible to adopt the simple subacromial deltoid space approach. The size of the calcaneal plate will be smaller after trimming based on the size of the fracture fragment, leading to no requirement for intraoperative exposure of the axillary nerve. Compared with the traditional proximal humeral locking plate, the calcaneal mesh plate can be used jointly with a variety of screws to fix the humeral head and humeral axis in multiple planes, capture separate fragments, and increase the stability of the structure. When the calcaneal mesh plate is fixed on the humeral greater tuberosity, it can contain the soft tissue and rotator cuff, just like a string bag, which can increase the stability of the structure in a way that only screws cannot achieve. For some patients with rotator cuff injury, it allows the suture for repairing the rotator cuff to be bound to the plate to strengthen the fixation, without impact on the blood supply of the rotator cuff (23, 24).

The present study showed that the calcaneal anatomical locking plate has the advantages of less surgical trauma, less bleeding, and better shoulder joint function. Additionally, this type of plate can reduce the incidence of shoulder impingement syndrome. Overall, the surgical technique of using calcaneal anatomical locking plate for the treatment of split fractures of humeral greater tuberosity is easy and efficient. The present study has some limitations. First, this study was performed based on a small sample size. Second, due to the short follow-up time, the implants were not completely removed in all patients at the last follow-up, which may produce a negative impact on the shoulder joint function. Third, biomechanical tests and finite element analysis of calcaneal plates for the treatment of split fractures of the humeral greater tuberosity will be performed in the future.

## Conclusion

Calcaneal anatomical locking plate may provide a new choice for the surgical treatment of split fractures of humeral greater tuberosity as it has the advantages of less surgical trauma, less bleeding, and better shoulder joint function.

## Data Availability

The raw data supporting the conclusions of this article will be made available by the authors, without undue reservation.
